# Role of N-acetylkynurenine in mediating the effect of gut microbiota on urinary tract infection: a Mendelian randomization study

**DOI:** 10.3389/fmicb.2024.1384095

**Published:** 2024-04-22

**Authors:** Yining He, Chao Han, Chengjuan Li, Xiaofan Yin, Jiawen Wang, Lina Gu, Ruxue Yan, Buhui Liu, Xuan Zhou, Weiming He

**Affiliations:** ^1^The First School of Clinical Medicine, Nanjing University of Chinese Medicine, Nanjing, China; ^2^Division of Nephrology, Affiliated Hospital of Nanjing University of Chinese Medicine, Jiangsu Province Hospital of Chinese Medicine, Nanjing, China; ^3^Yancheng Dafeng Hospital of Chinese Medicine, Teaching Hospital of Nanjing University of Chinese Medicine, Yancheng, China; ^4^Department of Human Anatomy, Xuzhou Medical University, Xuzhou, China; ^5^Department of Respiratory, The Fourth Affiliated Hospital of Nanjing Medical University, Nanjing, China

**Keywords:** gut microbiota, urinary tract infection, blood metabolites, N-acetylkynurenine, Mendelian randomization analysis

## Abstract

**Introduction:**

This study explored the causal connections between gut microbiota (GM), urinary tract infection (UTI), and potential metabolite mediators using Mendelian randomization (MR).

**Methods:**

We utilized summary statistics from the most comprehensive and extensive genome-wide association studies (GWAS) available to date, including 196 bacterial traits for GM, 1,091 blood metabolites, 309 metabolite ratios, alongside UTI data from ukb-b-8814 and ebi-a-GCST90013890. Bidirectional MR analyses were conducted to investigate the causal links between GM and UTI. Subsequently, two MR analyses were performed to identify the potential mediating metabolites, followed by a two-step MR analysis to quantify the mediation proportion.

**Results:**

Our findings revealed that out of the total 15 bacterial traits, significant associations with UTI risk were observed across both datasets. Particularly, taxon *g_Ruminococcaceae UCG010* displayed a causal link with a diminished UTI risk in both datasets (ukb-b-8814: odds ratio [OR] = 0.9964, 95% confidence interval [CI] = 0.9930–0.9997, *P* = 0.036; GCST90013890: OR = 0.8252, 95% CI = 0.7217–0.9436, *P* = 0.005). However, no substantial changes in *g_Ruminococcaceae UCG010* due to UTI were noted (ukb-b-8814: β = 0.51, *P* = 0.87; ebi-a-GCST90013890: β = −0.02, *P* = 0.77). Additionally, variations in 56 specific metabolites were induced by *g_Ruminococcaceae UCG010*, with N-acetylkynurenine (NAK) exhibiting a causal correlation with UTI. A negative association was found between *g_Ruminococcaceae UCG010* and NAK (OR: 0.8128, 95% CI: 0.6647–0.9941, *P* = 0.044), while NAK was positively associated with UTI risk (OR: 1.0009; 95% CI: 1.0002–1.0016; *P* = 0.0173). Mediation analysis revealed that the association between *g_Ruminococcaceae UCG010* and UTI was mediated by NAK with a mediation proportion of 5.07%.

**Discussion:**

This MR study provides compelling evidence supporting the existence of causal relationships between specific GM taxa and UTI, along with potential mediating metabolites.

## 1 Introduction

Urinary tract infection (UTI), a prevalent inflammatory condition affecting the urinary system, is primarily caused by uropathogenic *Escherichia coli* (UPEC) (Ji, 2005). The characteristics of the pathogen include high rates of incidence and recurrence, and increased antibiotic resistance (Tandogdu and Wagenlehner, 2016; Medina and Castillo-Pino, 2019; Faine et al., 2022). More than 50% of women experience at least one UTI during their lifetime (Foxman, 2014). Following antibiotic treatment, 20–30% of women experience a relapse within six months (Foxman, 2002), with approximately half of the recurrent strains derived from the initial infection (Silverman et al., 2013). Antibiotics, while effective in treating UTI, can also increase the risk of recurrence, potentially by causing disruptions in the GM (Köves et al., 2017). The existing understanding of its pathogenesis primarily focuses on the ascending infection pathway of intestinal bacteria. Pathogenic bacteria are excreted from the intestines into the feces, colonize around the urethra or vagina, and then ascend through the urethra to the bladder, triggering an infection. In addition, it is closely associated with factors such as mucosal immunity, estrogen levels, bacterial fimbriae and virulence factors. However, it remains unclear whether additional mechanisms exist through which gut bacteria can influence the recurrence of urinary tract infections.

Growing evidence suggests that the interaction between the gut and bladder, known as the gut-bladder axis, plays a crucial role in the pathogenesis of UTI (Yang et al., 2022). The gut-bladder axis implies that individuals with UTI often show an imbalance in the GM composition (Vervoort et al., 2015; Paalanne et al., 2018), and alterations in the GM can enhance susceptibility to recurrent UTI. Studies have revealed that uropathogens from the gut can repeatedly enter the urethra (Lee et al., 2014), and an increased abundance of pathogenic bacteria is a risk factor for bacteriuria after kidney transplantation (Magruder et al., 2019). Nevertheless, Worby et al. (2022b) have yielded conflicting conclusions, they discovered the influencing factor of recurrent UTI is not necessarily an increase in uropathogenic abundance, but rather a decrease in the diversity and richness of the GM. Nonetheless, owing to the influence of antibiotics and diet, it is challenging to ascertain whether dysbiosis of the GM is a consequence of UTI or an indicator of increased disease susceptibility.

The concept of gut-bladder axis also suggests that gut microbiota indirectly influence the host immune system through its metabolites, thereby exacerbating inflammation in the distal bladder (Worby et al., 2022a). For example, short-chain fatty acids (SCFAs) can modulate immune cell function and enhance the integrity of the intestinal barrier, thereby reducing the risk of urinary tract infections (Martin-Gallausiaux et al., 2021). Trimethylamine-N-oxide (TMAO) promotes the release of inflammatory factors during infection and increases the pathogenicity of *E. coli* in bladder cells (Wu et al., 2023). Iron participates in the replication of *E. coli* and in the host’s nutritional immune defense (Butler-Laporte et al., 2023). In addition, blood metabolites such as serum procalcitonin (Pecile et al., 2004), C-reactive protein (Shaikh et al., 2020), and serum calcitonin gene-related peptide (Lamot et al., 2022), play a crucial role in clinical diagnosis, which can be used as biomarkers for acute pyelonephritis in children. However, our current understanding does not clarify how the interaction between the GM and metabolites influences susceptibility to recurrent urinary tract infections.

Mendelian randomization (MR) analysis uses genetic variation as instrumental variables (IVs) to investigate the causal relationship between exposure and outcome. This method offers better control over confounding factors and sample size compared to clinical research, while also being more cost-effective. Moreover, to assess the causal relationship between GM and UTI, as well as the potential role of blood metabolites in this relationship, this study utilized statistical data from genome-wide association studies (GWAS) for MR analysis to clarify their relationship. Our study attempted to identify specific genera of bacteria or blood metabolites that may influence the occurrence of UTI, providing new perspectives for further mechanistic research, clinical diagnosis, and drug treatment.

## 2 Materials and methods

### 2.1 Overall design

**FIGURE 1:**
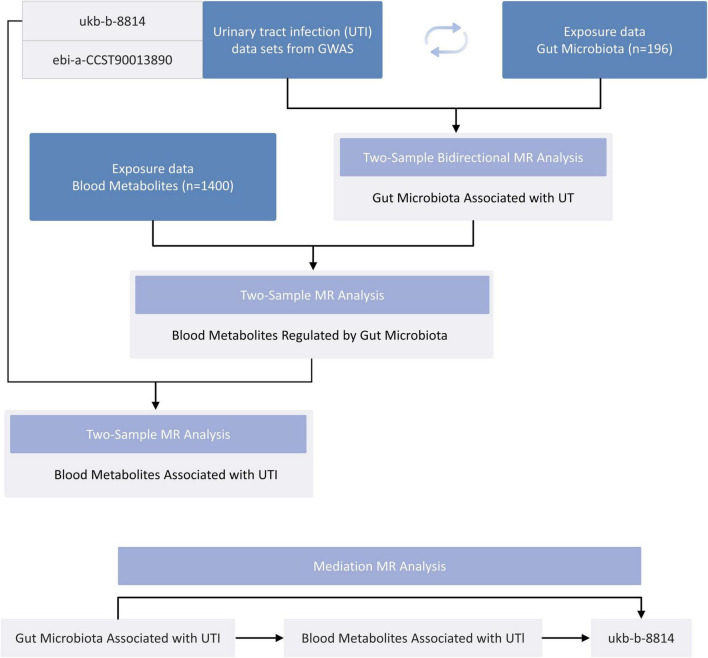
A flowchart detailing the Mendelian randomization study design.

The overarching design of the proposed study is illustrated in Figure 1. This investigation examined the bidirectional causal association between GM and UTI using a two-sample Mendelian randomization (TSMR) approach. To unravel the potential underlying mechanisms, we performed MR analyses to examine the relationship between the GM and serum metabolites. Subsequently, another MR analysis was carried out to explore the link between serum metabolites and UTI. To further probe the causal pathway from gut microbiota to UTI, a two-step MR design was employed for mediation analysis, specifically to investigate whether serum metabolites act as mediators in this pathway.

### 2.2 Data sources

Genetic variants correlated with the GM in this study were sourced from the MiBioGen Consortium’s extensive GWAS meta-analysis (van der Velde et al., 2019; Kurilshikov et al., 2021), encompassing 18,340 individuals across 24 cohorts, primarily of European descent (*n* = 13,266). GWAS data on tract infection were acquired by the publicly available GWAS catalog (ebi-a-GCST90013890) (Mbatchou et al., 2021) and the UK Biobank (ukb-b-8814). Summary-level statistical data for 1,091 blood metabolites and 309 metabolite ratios were derived from a comprehensive meta-analysis of the GWAS genomic atlas of the plasma metabolome, which prioritizes the metabolites implicated in human diseases (Chen et al., 2023). The data sources are presented in Table 1. Given that our study relied on publicly available summary data, there was no extra need for further ethical approval or consent procedures.

**TABLE 1:** Details of the genome-wide association studies and datasets utilized in our analyses.

Trait	GWAS ID	Consortium	Sample size	Year
Urinary tract infection	ukb-b-8814	MRC-IEU	463010	2018
Urinary tract infection	ebi-a-GCAT90013890 aaZGGCST90013890	NA	397867	2021
Human gut microbiome	NA	MiBioGen	18340	2021
1,091 blood metabolites and 309 metabolite ratios	NA	CLSA	8299	2023

### 2.3 TSMR design

**FIGURE 2:**
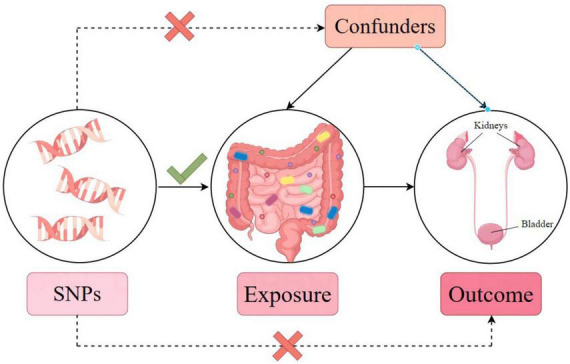
Mendelian randomization model and three key assumptions of a Mendelian randomization analysis.

In our analysis, single nucleotide polymorphisms (SNPs) are IVs. The three core assumptions of MR that must hold, to strengthen causal inference claims, are as follows (refer to Figure 2; Emdin et al., 2017): (1) Relevance: the genetic variants (SNPs) used as IVs must be associated with the exposure of interest. The selection criteria detailed below directly address this assumption by ensuring that only significantly associated SNPs are selected. (2) Independence: the IVs must not be connected to any confounders of the exposure-outcome relationship. The use of SNPs as IVs inherently supports this assumption, since genetic variants are randomly assorted at conception, independent of confounders that may affect the outcome studied later in life. (3) Exclusivity: the IVs influence the outcome exclusively through their association with the exposure, not through other pathways. The methodology employs various statistical methods for sensitivity analysis to detect and adjust for pleiotropy, supporting the exclusivity assumption. Furthermore, the robustness of the causal inference claims is further supported by multiple MR methods (inverse variance-weighted [IVW], MR-Egger regression, weighted median, simple mode, and weighted mode) to assess causal associations from different angles, providing a comprehensive view of the causal effect. Sensitivity analyses, including Cochran’s Q test for heterogeneity, MR-Egger intercept for horizontal pleiotropy, and leave-one-out analysis, were performed to detect and correct for potential biases or influential outliers. Through this streamlined methodology and assumption verification, we aimed to enhance the credibility and accuracy of our causal inference, providing robust methodological backing for our conclusions.

### 2.4 Selection of IVs

To obtain qualified IVs, the corresponding selection criteria were utilized. Initially, IVs were selected relying on a significance level of *P* < 1.0 × 10^(–5)^. This threshold ensures that only SNPs strongly associated with the exposure are considered, reducing the risk of selecting IVs by chance. Additionally, a linkage disequilibrium (LD) threshold of *R*^2^ < 0.001 and a clumping distance of 10,000 kb were applied using 1000 Genomes EUR data. This step minimizes the likelihood of selecting SNPs that are in LD with each other, ensuring that each SNP represents an independent source of genetic variation. Then, F-statistics were calculated to verify the robust connection between SNPs with an F-statistic of > 10 were considered to have a significant association with the exposure (Palmer et al., 2012; Gill et al., 2019; Levin et al., 2020). This criterion ensures that the selected IVs are strong instruments, reducing the risk of weak instrument bias, which can invalidate MR assumptions and conclusions.

### 2.5 Statistical analysis

This study employed multiple methodologies, including IVW (Burgess et al., 2015), MR-Egger regression (Bowden et al., 2015), weighted median (Bowden et al., 2016), simple mode, and weighted model (Hartwig et al., 2017), to assess causal associations. The IVW method is considered the primary analysis method due to its ability to offer accurate effect estimates and being commonly adopted as the predominant approach in the majority of MR analyses.

Diverse methods have been introduced for sensitivity analysis. First, Cochran’s Q test was used to assess IV heterogeneity, with a *P*-value of > 0.05 indicating no heterogeneity (Burgess et al., 2015). Second, the MR-Egger intercept method was used to quantify the heterogeneity effects among the genetic instruments. A *P*-value of less than 0.05, indicating a potential bias in the IVW estimate, could be attributed to horizontal pleiotropy. Additionally, MR-PRESSO detected anomalies and possible horizontal pleiotropy with a global *P*-value of less than 0.05, suggesting the existence of horizontal pleiotropy (Verbanck et al., 2018). Third, a leave-one-out sensitivity test identified potential heterogeneous SNPs (Burgess et al., 2017). Finally, funnel and forest plots were created for the direct identification of pleiotropy.

Statistical analyses were conducted using R version 4.2.1 (R Foundation for Statistical Computing, Vienna, Austria). The MR analyses were conducted using the TSMR (version 0.5.7) and MR-PRESSO (version 1.0) R packages (Hemani et al., 2018; Verbanck et al., 2018).

## 3 Results

### 3.1 Gut microbiota association with UTI through two-sample bidirectional MR analysis

**FIGURE 3:**
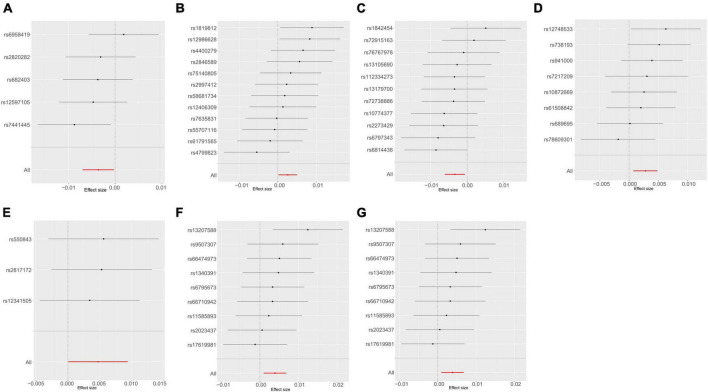
Mendelian randomization analyses show causal effects of gut microbiota on urinary tract infection using the ukb-b-8814 dataset. **(A–G)** Forest plots of 7 bacterial traits with *P*-value < 0.05 by IVW method. **(A)** genus *Ruminococcaceae* UCG010; **(B)** genus *Ruminococcus2*; **(C)** class Clostridia; **(D)** family Bacteroidales S24 7group; **(E)** genus *Clostridium* sensu stricto1; **(F)** genus *Bacteroides*; **(G)** family *Bacteroidaceae*.

A thorough evaluation was conducted within the framework of MR analysis, and the outcomes were visually represented in a heatmap (Gu et al., 2014; Supplementary Figure 1). The analysis revealed significant associations between seven bacterial traits across diverse taxonomic levels and UTI risk in the UKB dataset (Figures 3A–G). Among these traits, *c_Clostridia* (odds ratio [OR] = 0.9966, 95% confidence interval [CI]: 0.9938–0.9993, *P* = 0.016) and *g_Ruminococcaceae UCG010* (OR = 0.9964, 95% CI: 0.9930–0.9997, *P* = 0.036) were identified as protective factors. In contrast, *g_Ruminococcus2* (OR = 1.0026, 95% CI: 1.0001–1.0051, *P* = 0.038), *f_Bacteroidales S24 7group* (OR = 1.0027, 95% CI: 1.0006–1.0047, *P* = 0.012), *g_Clostridium sensu stricto1* (OR = 1.0047, 95% CI: 1.0000–1.0094, *P* = 0.048), and *g_Bacteroides* or *f_Bacteroidaceae* (OR = 1.0039, 95% CI: 1.0010–1.0068, *P* = 0.009, for both) were identified as risk factors. The detailed results are presented in Supplementary Table 1. Importantly, the *P*-values for pleiotropy and heterogeneity analyses consistently exceeded 0.05, indicating the absence of significant heterogeneity or pleiotropy issues. Moreover, these results were considered reliable and were supported by sensitivity analyses, which ruled out pleiotropy concerns.

**FIGURE 4:**
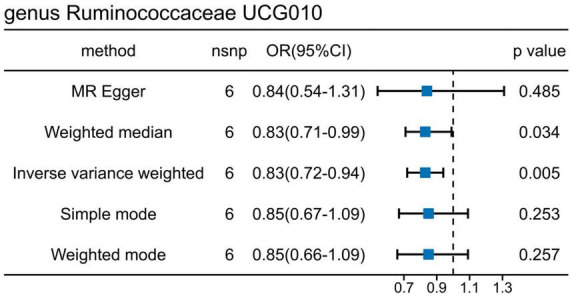
Mendelian randomization analyses show causal effects of *g_Ruminococcaceae* UCG010 on urinary tract infection using the ebi-a-GCST90013890 dataset.

The causal effects of the GM on UTI were investigated further using an additional dataset (ebi-a-GCST90013890) (Supplementary Figure 2), Using the IVW method, one order and two genera exhibited positive associations, whereas six genera showed negative associations with UTI (Supplementary Table 2). Interestingly, *g_Ruminococcaceae UCG010* was the only bacterial trait that overlapped between the two sets of results (Figure 4), indicating the potential involvement of specific bacterial traits in the onset of UTI.

Additionally, reverse MR analysis was conducted. Notably, *g_Ruminococcaceae UCG010* exhibited no significant changes attributed to UTI (ukb-b-8814: β = 0.51, *P* = 0.87; ebi-a-GCST90013890: β = −0.02, *P* = 0.77). Furthermore, no further evidence of a causal effect for UTI on the other taxa identified in the above results. When analyzing the ukb-b-8814 dataset, eight features (one class, one order, one family, and five genera) were significantly regulated. Similarly, the analysis of the ebi-a-GCST90013890 dataset revealed seven features with evident changes, including one family, and six genera.

### 3.2 Regulation of multiple metabolites by *Ruminococcaceae* UCG010

Recognizing the potential significance of metabolites in the interplay between GM and UTI, we conducted extensive MR analysis to unravel their intricate relationships. We discovered that *Ruminococcaceae UCG010* co-regulates 56 specific metabolites. Among these, 24 metabolites showed an upward regulatory trend and 32 showed a downward trend. Noteworthy metabolites, including sulfate of piperine metabolite C18H21NO3 (*P* = 0.0040), 1-(1-enyl-palmitoyl)-2-oleoyl-GPE (*P* = 0.0029), 9,10-DiHOME (*P* = 0.0061), 13-HODE + 9-HODE (*p* = 0.0087), N-palmitoyl-sphinganine (*P* = 0.0049), and 12,13-DiHOME (*P* = 0.0093), displayed a significant positive correlation with *Ruminococcaceae UCG010*. In contrast, Pseudouridine (*P* = 0.0058), 2-hydroxy-3-methylvalerate (*p* = 0.0033), and N2, N5-diacetylornithine (*P* = 0.0036) were significantly negatively correlated with *Ruminococcaceae UCG010*. Further details of the remaining metabolites are displayed in Supplementary Table 3.

### 3.3 Association of N-acetylkynurenine (NAK) with increased risk of UTI

**FIGURE 5:**
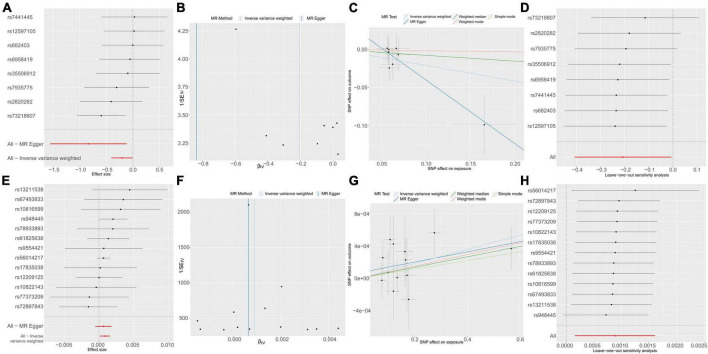
Causal effects of *g_Ruminococcaceae* UCG010 on N-acetylkynurenine **(A–D)** and N-acetylkynurenine on UTI **(E–H)**. **(A)** Forest plot. **(B)** Funnel plot to assess heterogeneity. **(C)** Scatter plots of genetic associations with *g_Ruminococcaceae* UCG010 against the genetic associations with N-acetylkynurenine. **(D)** Leave-one-out sensitivity analysis of SNPs associated with *g_Ruminococcaceae* UCG010 and their risk of N-acetylkynurenine. **(E)** Forest plot. **(F)** Funnel plot to assess heterogeneity. **(G)** Scatter plots of genetic associations with N-acetylkynurenine against the genetic associations with UTI. **(H)** Leave-one-out sensitivity analysis of SNPs associated with N-acetylkynurenine and their risk of UTI.

To delve into the relationship of causality between the 56 metabolites and UTI, we conducted MR analysis using the GWAS dataset (ukb-b-8814). IVW analysis revealed that only NAK acid (OR: 1.0009, 95% CI: 1.0002–1.0016, *P* = 0.0173) was significantly associated with an increased risk of UTI (Figure 5). This finding suggests that the increased risk of UTI associated with elevated levels of *Ruminococcaceae UCG010* may be partly attributed to the upregulation of NAK.

### 3.4 Mediation analysis of gut microbiome, NAK, and UTI

To explore the mediating role of N-acetylkynurenine, we calculated its indirect effects and proportions. The overall effect size was indicated by the β value between *Ruminococcaceae UCG010* and UTI, determined through TSMR. The indirect effect was derived by multiplying the β value of *Ruminococcaceae UCG010* to NAK with the β value of NAK to UTI. The direct effect was calculated by subtracting the indirect effect from the overall effect. Following these computations, the mediation effect manifested as −0.00018 (95% CI: −0.0419–0.0415), with a mediated proportion of 5.07%.

## 4 Discussion

In light of our awareness, this study emerges as the primary instance of being the first to delve deeply into the potential causal association between GM, blood metabolites, and urinary tract infections. Additionally, for the first time, we discovered a negative correlation between N-acetylkynurenine and *Ruminococcaceae UCG010*. These findings suggest that *Ruminococcaceae UCG010* plays a protective role against UTI and that the onset of UTI does not alter the abundance of this specific bacterial group. Our observations identified 56 metabolites associated with *Ruminococcaceae UCG010*. Further analysis revealed that this bacterium downregulated N-acetylkynurenine levels, subsequently contributing to UTI development. This finding provides theoretical support for the existence and mechanism of the gut-bladder axis from a genetic perspective.

In our study, the genus *Ruminococcaceae UCG010*, a member of the *Ruminococcaceae* family, emerged as a potential risk factor for UTI. Consistent with prior studies (Worby et al., 2022b), the abundance of *Ruminococcaceae* decreased in patients with recurrent UTI, suggesting that *Ruminococcus* could serve as a potential marker for dysbiosis in recurrent cystitis (Graziani et al., 2022). The *f_Ruminococcaceae* is also known to be significantly affected by chronic infections (Martinez et al., 2022; Tran et al., 2023) and the administration of antibiotics (Ross et al., 2016). Although direct evidence linking *Ruminococcaceae UCG010* to UTI remains elusive, some studies have proposed that a higher abundance of this bacterium in the GM may correlate with decreased risks of type 2 diabetes (Lyu et al., 2023), non-alcoholic fatty liver disease (Tsai et al., 2020), and obesity (Nseir et al., 2015), each being a risk factor for UTI. The mechanism involved in UTI could be that *Ruminococcaceae UCG010* produces SCFAs (Balmer et al., 2020). Hess discovered that acetate accumulates at the site of infection and regulates the inflammatory process by encoding the metabolic and functional reshaping of memory CD8+ T cells. Supplementation with acetate improved the symptoms of bladder inflammation and immune suppression caused by UPEC infection (Sturov et al., 2022). The production of butyrate in the intestine promotes the differentiation of Tregs, enhances epithelial barrier integrity, and inhibits proinflammatory responses (Siddiqui and Cresci, 2021). Decreased butyrate abundance is connected to heightened susceptibility to infections (He et al., 2022). In addition, *Ruminococcaceae* are involved in bile acid (BA) metabolism (Pickard et al., 2017), they can decompose cholesterol, generate secondary bile acids, and influence the formation of urinary stones (Zhou et al., 2023). A humanized dyslipidemia mouse model demonstrated that *Ruminococcaceae UCG010* reduced BA synthesis (Xu et al., 2023). Interestingly, we also discovered that *Ruminococcus2*, which belongs to the *Ruminococcaceae* family, is a risk factor for UTI. This indicates that despite their taxonomic similarity, different genera or species within the same family can have different functions and characteristics. These factors may be influenced by the genomic and metabolic capabilities. Further experiments and observations are required to obtain a more detailed understanding of the functions of these microorganisms and their effects on human health.

As a major degradation product of N-acetyl tryptophan (Agus et al., 2018), NAK has been found to be an activator of aryl hydrocarbon receptor (AhR) (Rael et al., 2018). NAK inhibits macrophage activation by activating AhR, thereby reducing the secretion of interleukin-6 (IL-6) and chemokine-10 (CXCL-10). Macrophages, IL-6, and CXCL-10 are closely related to inflammatory responses. M1 macrophages primarily mediate tissue damage and initiate inflammatory responses, whereas M2 macrophages principally inhibit granulation and scar formation (Han et al., 2019; Kuhn et al., 2023). Transition from M1 to M2 can be observed during the progression of acute to chronic urinary tract infection (Hreha et al., 2020). However, premature inhibition of M1 could compromise the bactericidal function of the bladder. IL-6 is an early marker that rapidly increases during inflammation (Rashid et al., 2021). Furthermore, IL-6 deficient mice demonstrated heightened UPEC load, decreased antimicrobial peptide release, and heightened mortality (Khalil et al., 2000). Clinical investigations have documented a considerable rise in CXCL-10 in the urine of patients with UTI, with levels decreasing following antibiotic administration. CXCL-10 drives inflammation by activating T cell chemotaxis and endothelial adhesion, as well as by enhancing cell lysis mediated by natural killer cells (Platten et al., 2019). This suggests that NAK may inhibit the body’s inflammatory response and thereby affect pathogen clearance.

Our mediation analyses further supported the genetic evidence for a link between GM and UTI. To the best of our knowledge, no prior study has directly linked *Ruminococcaceae UCG010* to N-acetylkynurenine. Furthermore, it is imperative to acknowledge the inherent limitations and potential sources of bias within MR studies, including our own. MR analyses rely on several key assumptions: the genetic variants employed as IVs are associated with the exposure but not with any confounders of the exposure-outcome relationship, and these variants influence the outcome solely through the exposure. While we have strived to select IVs with strong associations and minimal linkage disequilibrium to mitigate pleiotropy and bias, residual confounding due to unmeasured or inadequately measured variables, and potential violation of these assumptions may still affect the interpretation of our findings. However, a growing number of research have explored the involvement of the GM in tryptophan metabolism pathways, including indole-uracil, serotonin, and aromatic amino acid metabolism pathways (Xue et al., 2023). Recently, the involvement of *Ruminococcus* species in tryptophan metabolism has been confirmed (Coletto et al., 2022). *Ruminococcaceae UCG010* is a major producer of SCFAs, and its metabolite butyrate can influence the activity of intestinal epithelial cells (IELs) indoleamine 2,3-dioxygenase (IDO) (Martin-Gallausiaux et al., 2018). During UPEC infection, local IDO levels in the bladder increase, promoting indole-uracil production, and inhibiting neutrophil chemotaxis (Loughman et al., 2016). Studies have shown that kynurenine, a product of Try degradation, can serve as a key signaling molecule to activate AhR and promote the Treg-macrophage axis in suppressing T-cell dysfunction (Campesato et al., 2020). Additionally, research has found associations between Try metabolites and infectious diseases. Kynurenines exhibit antimicrobial activity and directly influence the proliferation of gut microbiota (Notarangelo et al., 2014). Experiments have demonstrated that the host’s AhR receptor can qualitatively and quantitatively perceive the quorum sensing signal molecules secreted by Pseudomonas aeruginosa at various stages of infection and coordinate host defense functions based on the infection status (Moura-Alves et al., 2019). N-acetylkynurenine is an AhR agonist. AhR participates in inflammatory responses and immune tolerance, regulates the mucosal barrier function, and maintains intestinal homeostasis (Su et al., 2022). Deficiency of the AhR repressor leads to fewer IELs, which can cause intestinal infection and inflammation (Gao et al., 2022). By virtue of our findings and previous literature, we speculate that *Ruminococcaceae UCG010* may reduce the production of N-acetyl-kynurenine through the tryptophan acid pathway, inhibiting AhR-mediated inflammatory responses in macrophages and other cells, thereby affecting the occurrence and progression of tract infections.

Our MR analysis revealed that more than 30 gut microbial taxa were causally associated with UTI. Similar to other studies, patients with UTI exhibit gut dysbiosis, such as decreased levels of *Lactobacillus* and *Bifidobacteria*, along with an increased abundance of conditionally pathogenic enterobacteria and *Clostridium* (Stepanova et al., 2018). However, most of the gut microbial taxa identified in our study have rarely been reported to be associated with UTI in previously published literatures. To investigate the mechanism underlying this causal relationship, we interpreted it from several perspectives. (1) An imbalance in the GM facilitates the colonization of pathogenic bacteria (Bowden et al., 2016), enhancing their adhesion and toxicity. During UPEC infection, the TLR4/NF-κB cell pathway is activated and the expression of the host cell phosphatase transporter protein Pituitary specific transcription factor 1 is upregulated. This mediates the escape of UPEC from the vacuoles to the cytoplasm, thereby evading a systemic immune response (Pang et al., 2022). (2) In this study, the bacterial genera that had causal relationships were mostly those that produced SCFAs, such as *Bacteroidales*, *Turicibacter*, and so on. The metabolic products of fatty acids produced by gut microbiota also exert direct antibacterial effects. They suppress the expression of virulence genes in *E. coli*, thereby decreasing the adhesion and motility of extraintestinal pathogenic *E. coli* (ExPEC). In contrast, SCFAs can freely diffuse inside and outside bacterial cell membranes, causing pH disturbances between cells and thus inhibiting bacterial growth (Buffie and Pamer, 2013). In patients with recurrent UTI, gut dysbiosis is often accompanied by a reduction in SCFAs levels. In particular, the absence of bacteria that produce butyrate, an important component of SCFAs, is notable (Worby et al., 2022b). Butyrate plays a critical role in maintaining the intestinal barrier integrity, improving intestinal inflammation, and promoting immune regulation (Sturov et al., 2022). Therefore, dysbiosis of the gut microbiota, which results in a decrease in SCFAs, may increase the risk of urinary tract infections. (3) Urinary tract infection is closely relevant to the intestinal barrier function, which is to prevent the entry of harmful substances, such as bacteria, toxins, and antigens, from the intestine into the bloodstream. ExPECs have been demonstrated to establish specific interactions with the epithelial barrier of the intestine (Poole et al., 2017), and to induce dysfunction of the intestinal barrier prior to the onset of disease (Nagpal and Yadav, 2017). antibiotic treatment can disrupt the structure and function of tight junction proteins, leading to tight junction dysfunction and increased intestinal permeability (Feng et al., 2019), which facilitates the translocation of antigens and toxic substances into systemic circulation, thereby contributing to the development of chronic inflammation (Sharapatov et al., 2021).

This study encompasses several limitations that warrant careful consideration for a comprehensive understanding of its scope and implications of its findings. Firstly, the GWAS analysis conducted leveraged data primarily derived from a European demographic, inherently limiting the extrapolation of our findings to diverse populations, including those characterized by distinct genetic compositions and environmental exposures. This demographic focus raises questions about the universality of our conclusions and underscores the imperative for inclusive research involving a broader spectrum of ethnicities to ensure global applicability. Secondly, the reliance on aggregated summary data for UTI cases in our study precluded the possibility of subgroup analyses, notably those differentiated by sex and age. This reliance significantly hampers our understanding of UTI dynamics across different demographics, as it masks potential variations in disease susceptibility and progression that are crucial for personalized medical management approaches. Thirdly, our investigation into the gut microbiome was restricted to the genus level, constrained by the taxonomic resolution provided in the GM dataset. This limitation prevented us from delving into the potentially more informative specie-level analysis, which could offer finer insights into microbiota-UTI interactions. The ability to analyze at the species level could reveal nuanced microbial behaviors and their specific roles in urinary tract infections, marking a critical area for future exploration. To address these limitations and advance our comprehension of the intricate interplay among GM, their metabolites, and UTI, future research should aim for a more inclusive and detailed approach. This includes expanding the genetic and environmental diversity of study populations, enhancing the taxonomic resolution of microbiome analysis, and implementing rigorous methods to control for confounders and biases. Furthermore, a more nuanced application and critical evaluation of MR methods are essential to refine our causal inferences. Such comprehensive efforts are pivotal for unraveling the intricate mechanisms underlying UTI and tailoring effective prevention and treatment strategies across varied population segments.

Previous clinical studies have revealed a correlation between GM and UTI. It is challenging to establish a causal relationship, due to various confounding factors. Our research finds that gut microbiota and blood metabolites can influence the onset and progression of UTI. With the increasing prevalence of antibiotic resistance, there is an urgent demand for novel approaches to treating UTI. Besides conventional treatments targeting pathogens, our study proposes a new investigation of possibility, namely the microbial therapy. Gut microbiota can serve as potential biological markers for UTI and directly affect their recurrence and prognosis. Focusing on gut microbiota and blood metabolites, we can delve into deeper mechanism studies and drug intervention clinical trials. Biological therapies such as fecal microbiota transplantation (FMT) and probiotics have initiated preliminary clinical studies, but these treatments possess limitations. FMT is a non-targeted therapy and commercially available probiotics consist mostly of single strains or a mixture of several strains, none of which were specifically developed for the characteristics of UTI pathology. Notably, our study identified protective commensal bacterial groups against UTI, paving the way for the development of personalized microbiome-based therapeutic strategies.

## 5 Conclusion

Our findings, obtained through mediation analysis, indicated that *Ruminococcaceae UCG010* can act as a direct protective agent against UTI and indirectly reduce the occurrence of UTI by reducing N-acetylkynurenine levels. Consequently, this study provides independent evidence supporting the association between the composition of the gut microbiota and the risk of developing UTI.

## Data availability statement

The original contributions presented in this study are included in the article/Supplementary material, further inquiries can be directed to the corresponding authors.

## Author contributions

YH: Conceptualization, Formal analysis, Funding acquisition, Investigation, Methodology, Project administration, Validation, Writing – original draft, Writing – review & editing. CH: Data curation, Formal analysis, Investigation, Methodology, Writing – review & editing. CL: Formal analysis, Investigation, Methodology, Validation, Writing – review & editing. XY: Data curation, Formal analysis, Writing – review & editing, Validation. JW: Formal analysis, Funding acquisition, Validation, Writing – review & editing. LG: Formal analysis, Writing – review & editing. RY: Formal analysis, Methodology, Writing – review & editing. BL: Formal analysis, Supervision, Writing – review & editing. XZ: Conceptualization, Data curation, Project administration, Writing – review & editing. WH: Conceptualization, Funding acquisition, Project administration, Supervision, Writing – review & editing.

## Supplementary material

Supplementary Figure 1 Heatmap of MR results.

Supplementary Figure 2 Mendelian randomization analyses show causal effects of gut microbiota on UTI using the ebi-a-GCST90013890 dataset.
