# Supplementary figures and images for: Role of N-acetylkynurenine in mediating the effect of gut microbiota on urinary tract infection: a Mendelian randomization study
Source: Front Microbiol. 2024 Apr 22;15:1384095. doi: 10.3389/fmicb.2024.1384095 (PMC11070472; doi:10.3389/fmicb.2024.1384095)

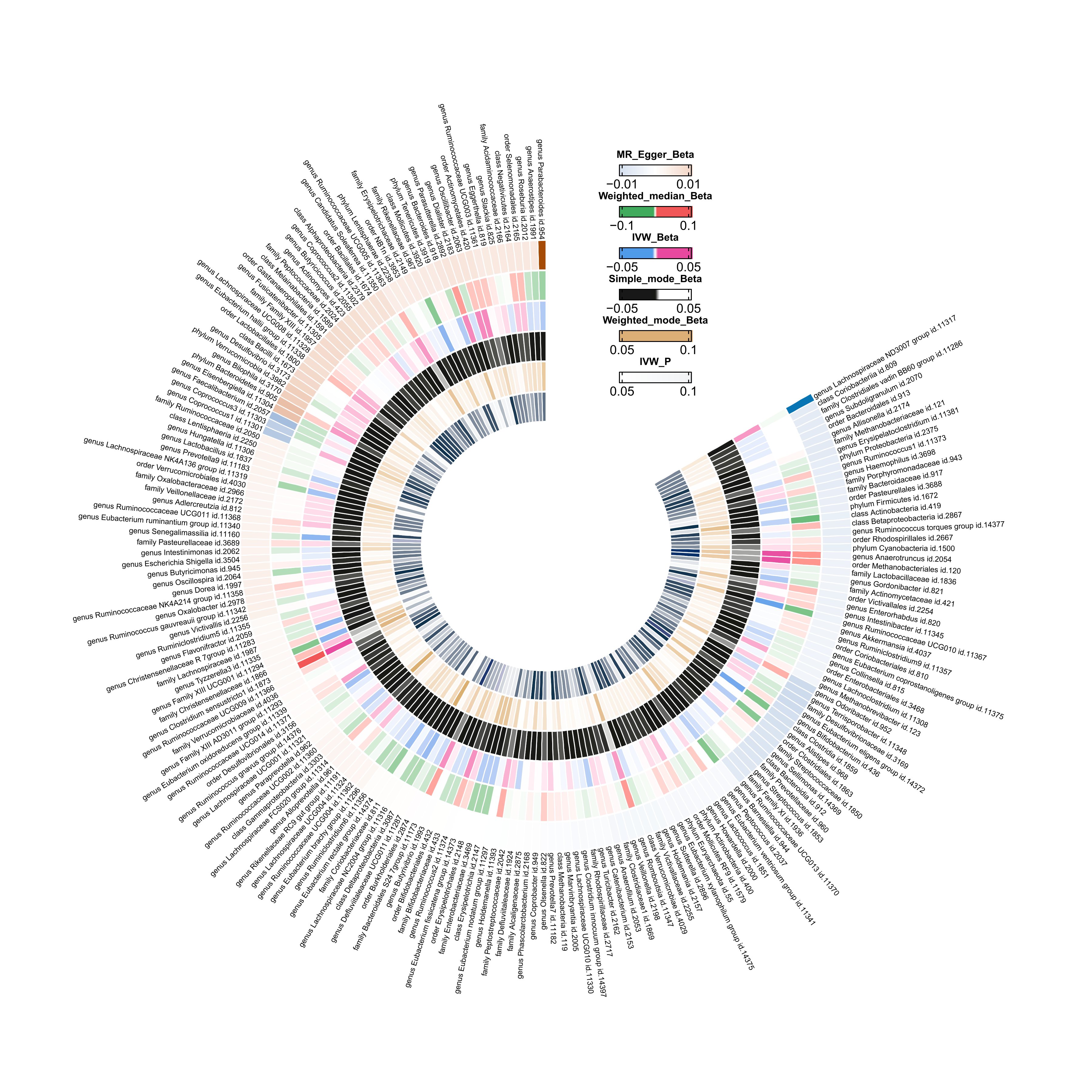

Supplement: Supplementary Figure 1 — Heatmap of MR results. [file Image_1.jpg]

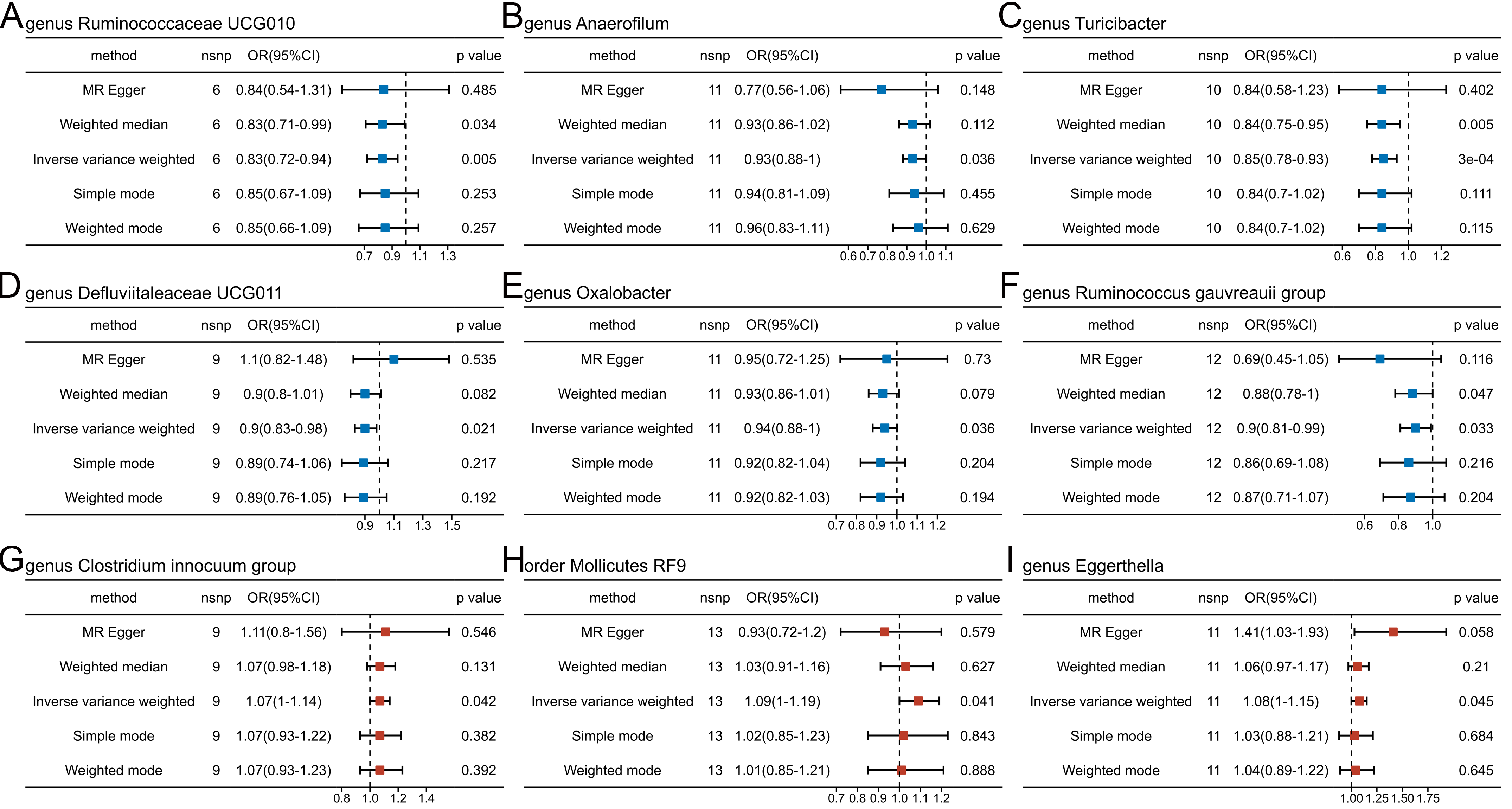

Supplement: Supplementary Figure 2 — Mendelian randomization analyses show causal effects of gut microbiota on UTI using the ebi-a-GCST90013890 dataset. [file Image_2.tif]
